# Subcellular localization of the mouse PRAMEL1 and PRAMEX1 reveals multifaceted roles in the nucleus and cytoplasm of germ cells during spermatogenesis

**DOI:** 10.1186/s13578-021-00612-6

**Published:** 2021-06-01

**Authors:** Wan-Sheng Liu, Chen Lu, Bhavesh V. Mistry

**Affiliations:** 1 Department of Animal Science, Center for Reproductive Biology and Health (CRBH), College of Agricultural Sciences, The Pennsylvania State University, 324 Henning Building, University Park, PA 16802 USA; 2 Present Address: Fudan University, Shanghai, People’s Republic of China; 3 Present Address: Department of Comparative Medicine, King Faisal Specialist Hospital & Research Centre, Riyadh, Saudi Arabia

**Keywords:** PRAMEL1, PRAMEX1, Cancer-testis antigen, Immunoelectron microscopy, Germ granule, Chromatoid body, Nucleocytoplasmic transport, Germ cell, Spermatogenesis, Testis

## Abstract

**Background:**

*Preferentially expressed antigen in melanoma* (PRAME) is a cancer/testis antigen (CTA) that is predominantly expressed in normal gametogenic tissues and a variety of tumors. Members of the *PRAME* gene family encode leucine-rich repeat (LRR) proteins that provide a versatile structural framework for the formation of protein–protein interactions. As a nuclear receptor transcriptional regulator, PRAME has been extensively studied in cancer biology and is believed to play a role in cancer cell proliferation by suppressing retinoic acid (RA) signaling. The role of the PRAME gene family in germline development and spermatogenesis has been recently confirmed by a gene knockout approach. To further understand how PRAME proteins are involved in germ cell development at a subcellular level, we have conducted a systematic immunogold electron microscopy (IEM) analysis on testis sections of adult mice with gene-specific antibodies from two members of the mouse *Prame* gene family: *Pramel1* and *Pramex1*. *Pramel1* is autosomal, while *Pramex1* is X-linked, both genes are exclusively expressed in the testis.

**Results:**

Our IEM data revealed that both PRAMEL1 and PRAMEX1 proteins were localized in various cell organelles in different development stages of spermatogenic cells, including the nucleus, rER, Golgi, mitochondria, germ granules [intermitochondrial cement (IMC) and chromatoid body (CB)], centrioles, manchette, and flagellum. Unlike other germ cell-specific makers, such as DDX4, whose proteins are evenly distributed in the expressed-organelle(s), both PRAMEL1 and PRAMEX1 proteins tend to aggregate together to form clusters of protein complexes. These complexes were highly enriched in the nucleus and cytoplasm (especially in germ granules) of spermatocytes and spermatids. Furthermore, dynamic distribution of the PRAMEL1 protein complexes were observed in the microtubule-based organelles, such as acroplaxome, manchette, and flagellum, as well as in the nuclear envelope and nuclear pore. Dual staining with PRAMEL1 and KIF17B antibodies further revealed that the PRAMEL1 and KIF17B proteins were co-localized in germ granules.

**Conclusion:**

Our IEM data suggest that the PRAMEL1 and PRAMEX1 proteins are not only involved in transcriptional regulation in the nucleus, but may also participate in nucleocytoplasmic transport, and in the formation and function of germ cell-specific organelles during spermatogenesis.

## Background

Spermatogenesis is a continuous process throughout the reproductive lifetime of mature males. It is a complex cellular transformation process within the seminiferous tubules of the testis, which can be divided into three functional phases: mitosis, meiosis and spermiogenesis. During spermiogenesis, haploid round spermatids undergo a dramatic morphological transformation. The nucleus of spermatids undergoes a profound shape change and condensation and the histone proteins are replaced by protamine [1], leading to the transcriptional shutdown of the genome. The acrosome develops and spreads over the anterior nucleus; centrioles move to the posterior nucleus to form the sperm tail; the excess cytoplasm is dropped and mitochondria are rearranged to the midpiece of sperm tail; finally, the morphologically distinct spermatozoa undergo spermiation and are released from Sertoli cells into the lumen of seminiferous tubules [2].

Germ granules (also known as nuage) are cytoplasmic, nonmembrane-bound organelles, which are described as various forms of dense material in differentiating germ cells of a wide variety of species [3, 4]. Two different types of germ granules, i.e. intermitochondrial cement (IMC) and chromatoid body (CB), are present in mammalian spermatogenic cells [5, 6]. IMC is found mainly in late spermatocytes, whereas CB is present in the cytoplasm of post-meiotic spermatids. CB moves around the nucleus in the cytoplasm and even passes through cytoplasmic bridges of sister spermatids; it contains coding and non-coding RNAs, and RNA-binding proteins involved in various RNA regulatory pathways [6, 7]. Many germ cell-specific proteins, such as DDX4 (DEAD-box helicase 4, also known as VISA, or MHV) [8–11], PIWIL1 (piwi like RNA-mediated gene silencing 1) [12, 13], TDRD1 (tudor domain containing protein 1) [14, 15] and MAEL (maelstrom spermatogenic transposon silencer) [16] are predominantly located in CB and are critical for germ cells development and function [17, 18]. Interestingly, a recent study revealed that the bovine PRAMEY (preferentially expressed antigen in melanoma, Y-linked) protein is present mainly in IMC and CB of spermatogenic cells [19]. In the present study, we found that the mouse PRAMEX1 (Prame, X-linked 1) and PRAMEL1 (Prame like 1) proteins are also located in IMC and CB, signifying the importance of the *PRAME* gene family in spermatogenesis.

PRAME was first discovered in 1997 as a tumor antigen in human melanoma cells [1]. As a cancer/testis antigen (CTA), PRAME is predominately expressed in the normal testis, as well as in a variety of tumors with functions in immunity and reproduction [1–4]. The *PRAME* gene has been amplified during evolution and constitutes a large gene family in eutherian mammals [20, 21]. There are > 30 PRAME paralogs of in the human, ~ 90 in mouse, and ~ 60 in the bovine genome [21]. Although the *PRAME* gene expansion occurred mainly among autosomes, *PRAME* copies have also translocated on to the sex chromosome (chr) in rodent (chr X) and bovid (chr Y) lineages [21]. The mouse *Prame* gene family is the third largest family in the genome, and maps on chromosome 2, 4 and X in large clusters [20–22]. Although the copy number variations (CNVs) of the human and mouse *PRAME* genes have not been studied, research on the bovine *PRAMEY* subfamily found that its CNVs are associated with testicular size and semen quality [23].

PRAME is a leucine rich repeat (LRR) protein that has a nuclear localization signal (NLS) [21, 24–27]. The basic three-dimensional (3D) structure of LRR proteins fold into a horseshoe shape, a conformation that provides a structural framework for protein–protein interactions [28]. Due to the flexibility of LRR domains, LRR proteins participate in many important biological processes, including hormone-receptor interactions, enzyme inhibition, cell adhesion and cellular trafficking [29].

As a dominant repressor, PRAME was involved in the retinoic acid receptor (RAR) signaling in melanoma and other cancer cells [30], though a later study indicated that PRAME was not associated with RAR signaling in primary acute myeloid leukemia [31] and seminomas [32]. Despite extensive studies of PRAME in cancer biology, few reports have focused on the function of PRAME in spermatogenesis. Our earlier study indicated that the mouse *Pramel1* and *Pramex1* are exclusively expressed in the testis [33], and deletion of *Pramex1* leads to a smaller testis and a significant reduction in sperm count [34]. Recent studies on gene-specific knockout (KO) mice revealed that *Pramef12* (Prame family 12) is required for spermatogonial stem cell (SSC) self-renewal and proliferation [35], while *Pramel7* (Prame like 7) and *Gm12794c* (a member of the mouse *Prame* family) function in embryonic stem cell (ESC) self-renewal and maintenance of pluripotency [36–38].

Although accumulating evidence suggests that the *PRAME* gene family is essential for germline development and spermatogenesis [39], the subcellular localization of the mouse PRAME proteins in germ cells has not been investigated. To fill this knowledge gap, we have performed a systematic immunogold electron microscopy (IEM) analysis on testis sections of adult mice with gene-specific antibodies from two members of the mouse *Prame* gene family: *Pramel1* (on chr 4) and *Pramex1* (on chr X). We first examined the protein localization patterns in different spermatogenic cells, with a focus on the dynamics of the PRAMEL1 protein complex in germ granules and other cellular organelles in different stages of spermatids. Then, we analyzed the co-localization patterns of PRAMEL1 and two other CB-enriched proteins, DDX4 and KIF17b (kinesin family member 17b), in germ granules. Our data strongly suggest that the mouse PRAMEL1 and PRAMEX1 proteins may play multifaceted roles in the nucleus and cytoplasm of germ cells during spermatogenesis.

## Materials and methods

### Animals

All animal procedures were performed in accordance with the Guide for the Care and Use of Laboratory Animals and approved by the Institutional Animal Care and Use Committee (IACUC) of the Penn State University (Protocol #46391). The inbred C57BL/6 mice were housed at the Transgenic Mice Core Facility, in the Huck Institutes of the Life Sciences, Penn State University, with free access to food and water under a 12 h (h) of light and 12 h of darkness cycle.

### Preparation of testicular tissues

Testis sections and squashed seminiferous tubules were prepared as described previously [33, 40]. Briefly, whole testes were dissected out of the adult mice and fixed by incubation overnight in Bouin solution (Sigma-Adrich, St. Louis, MO) at 4 °C. Tissues were then washed in 70% ethanol, dehydrated, and embedded in paraffin blocks. The paraffin-embedded tissues were sectioned at 4 µm thickness using a microtome and mounted on glass slides. To obtain squashed seminiferous tubules, adult testes were excised and transferred to a glass Petri dish containing ice-cold PBS (140 mM NaCl, 2.6 mM KCl, 6.4 mM Na_2_HPO_4_, 1.4 mM KH_2_PO_4_, pH 7.4). After removing the tunica albuginea, seminiferous tubules were transferred to a new Petri dish containing PBS. Using fine forceps, the tubules were gently pulled apart and a small piece of single tubule was cut. The piece of tubule was transferred into a 15–30 ul drop of 0.1 M sucrose solution prepared in PBS on a new Petri dish. The cells were released from the tubule by tweezing apart the tubule and resuspending it in the sucrose solution by pipetting up and down. Subsequently, the cells were transferred to slides predipped in 1% paraformaldehyde and 0.15% Triton-X100. Slides were dried and processed for immunofluorescence staining or frozen at − 80 °C for later use.

### Isolation of epididymal sperm

Spermatozoa were collected by mincing caput, corpus and cauda epididymal sections from adult mice into 37 °C-equilibrated PBS for 5 min. Sperm were washed by centrifugation (300×*g*, 5 min) and resuspended in cold 0.1 M sucrose solution in PBS. Sperm smears were prepared on slides predipped in 1% paraformaldehyde and 0.15% Triton-X100. Slides were dried at room temperature (RT) and processed for immunofluorescence staining as described below or stored at − 80 °C for later use [33].

### Indirect immunofluorescence of testes and spermatozoa

Testis cross-section preparation and immunofluorescence (IF) staining on testis sections and sperm cells were performed using a previously described protocol [33]. The primary anti-PRAMEL1 antibody (termed as PRAME like-1 (S-16), sc-34513) was purchased from Santa Cruz Biotechnology (Santa Cruz, CA). This antibody is an affinity-purified goat polyclonal antibody raised against a peptide of PRAMEL1 of the mouse protein (accession no. NP_113554), which was chosen from the C-terminal region between 350 and 400 amino acid (aa). The primary anti-PRAMEX1 (also known as PRAME) antibody raised in rabbit against the N-terminal of the human PRAME was purchased from Aviva company (ARP55982_P050), with a 92% similarity to the mouse homology.

The primary antibodies PRAMEL1 and β-TUBULIN (Sigma-Adrich, T4026) were diluted 1:50 in PBST, and the secondary antibodies FITC-conjugated donkey anti-goat IgG and TRITC-conjugated donkey anti-mouse IgG (both diluted 1:100 in PBST) were applied, respectively. Cell nuclei were counterstained with DAPI and the coverslips were mounted as described above for testis cross-sections. All the samples were observed at RT using Olympus BX51 (Olympus Optical Co. Ltd, Tokyo, Japan) epifluorescence microscope and digital images were captured with an Olympus DP71 microscope camera (Olympus America Inc., Center Valley, PA). Adobe Photoshop software (Adobe Inc., San Jose, CA) was used to assemble images into figures. No post-acquisition modifications were made to the original images.

### Sample processing for transmission electron microscopy (TEM)

Adult mice were perfused with PBS via the heart until solution coming out of the heart was clear and the liver looked pale, at which time perfusion was switched to 4% paraformaldehyde (PFA). Testes were then cut into small pieces (~ 5 mm) and put in fresh fixative solution (4% PFA + 0.1% glutaraldehyde, GA) for 2 h at RT. After that, the testis samples were transferred into fresh fixative solution and placed in a 4 °C refrigerator for at least 3 h. The testis samples were then transferred into 2.3 M sucrose and incubated overnight at 4 °C.

The infiltrated sample was then frozen in liquid nitrogen followed by the freeze-substitution procedures performed in Automatic Freeze Substitution System as described previously [19]. Great care was taken to prevent warming the specimen following this step. The frozen samples were transferred to 0.5% uranyl acetate in methanol which was kept at − 90 °C in the freeze-substitution unit. Five hours later, most of the solution was withdrawn and fresh methanol/0.5% uranyl acetate solution was added. The temperature was raised to − 80 °C for 24 h. The specimens were then rinsed three times with pure methanol at − 70 °C over a period of 8 h, and three times with pure methanol at − 45 °C over a period of 20 h. The samples were infiltrated at 45 °C, with 1:1 mixture of methanol and Lowicryl HM20 (EMS company, Catalog#14340) for 6 h, 1:2 mixture of methanol and Lowicryl HM20 for 14 h, pure Lowicryl HM20 for 8 h with three changes, and pure Lowicryl HM20 for 24 h. The samples were changed to fresh Lowicryl HM20 and polymerized with indirect UV light at − 45 °C for 48 h and 0 °C for another 24 h. After complete polymerization, the specimen was taken to RT and sectioned to 70 nm ultrathin sections using conventional ultramicrotome followed by placing on a nickel grid for examination (Microscopy and Cytometry Facility, Huck Institutes of the Life Sciences, Penn State University).

### Immunoelectron microscopy (IEM)

The testis sections on nickel grids were briefly rinsed in PBS and then incubated for 5 min in 1% glycine solution prepared in 1X PBS. The sections were blocked in 1% BSA/PBS for 30 min, followed by 2.5 h incubation of anti-PRAMEL1 antibody (Santa cruz 1:50 dilution, ~ 1.5 µg/ml) or PRAMEX1 antibody (1:50 dilution, ~ 1.5 µg/ml). The same concentration (~ 1.5 µg/ml) of goat IgG or rabbit IgG served as negative controls for PRAMEL1 and PRAMEX1 IEM, respectively. After the primary antibody incubation, the grids were washed 5 times (3 min each) with PBS and then incubated with the secondary antibody, donkey anti-goat IgG or donkey anti-mouse IgG (both diluted 1:100 in PBS) that was conjugated with gold particles (5 or 10 nm) for 1 h at RT. Before drying, the grids were washed 5 times in PBS and 7 times in ultrapure water. The grids were examined by a transmission electron microscope (JEOL1200, at Microscopy and Cytometry Facility, Huck institutes of the Life Sciences, The Pennsylvania State University).

Large clusters of PRAMEL1 gold particles were observed in the cellular organelles of germ cells in a pilot study. To determine whether these clusters were caused by the secondary antibody aggregation, we performed the following 4 groups of IEM experiments: (I) PRAMEL1 + secondary antibody without centrifugation; (II) PRAMEL1 + secondary antibody with centrifugation (supernatant); (III) PRAMEL1 + secondary antibody with centrifugation (bottom); and (IV) Negative control (secondary antibody without centrifugation). A total of 30 ul of the secondary antibody were centrifuged at 10,000 rpm for 10 min. Then the top 5 μl of supernatant were transferred into a new tube for Group II and the very bottom 5 μl of the centrifuged secondary antibody were used for Group III. The incubation time and washing conditions for the secondary antibody were the same as described above.

For the co-localization of PRAMEL1 and DDX4 (Abcam, ab13840, 1:50 dilution) or PRAMEL1 and KIF17b (Aviva Systems Biology, ARP57461_P050, 1:50 dilution) the basic protocol was the same as the single antibody PRAMEL1 IEM analysis except for the application of different sizes of gold particles conjugated in the secondary antibody that would allow us to identify the location of the two different proteins on the dual-strained sections. In brief, the anti-PRAMEL1 antibody and the second primary antibody, either anti-DDX4, or anti-KIF17b, were mixed in 1% BSA/PBS before applying to the grids for the incubation process. The secondary antibody for PRAMEL1 was conjugated with 10 nm gold particles, while those against DDX4 or KIF17b antibody were conjugated with 5 nm gold particles.

## Results

### Cellular localization of the mouse PRAMEL1 protein in spermatids and spermatozoa

**Fig. 1:**
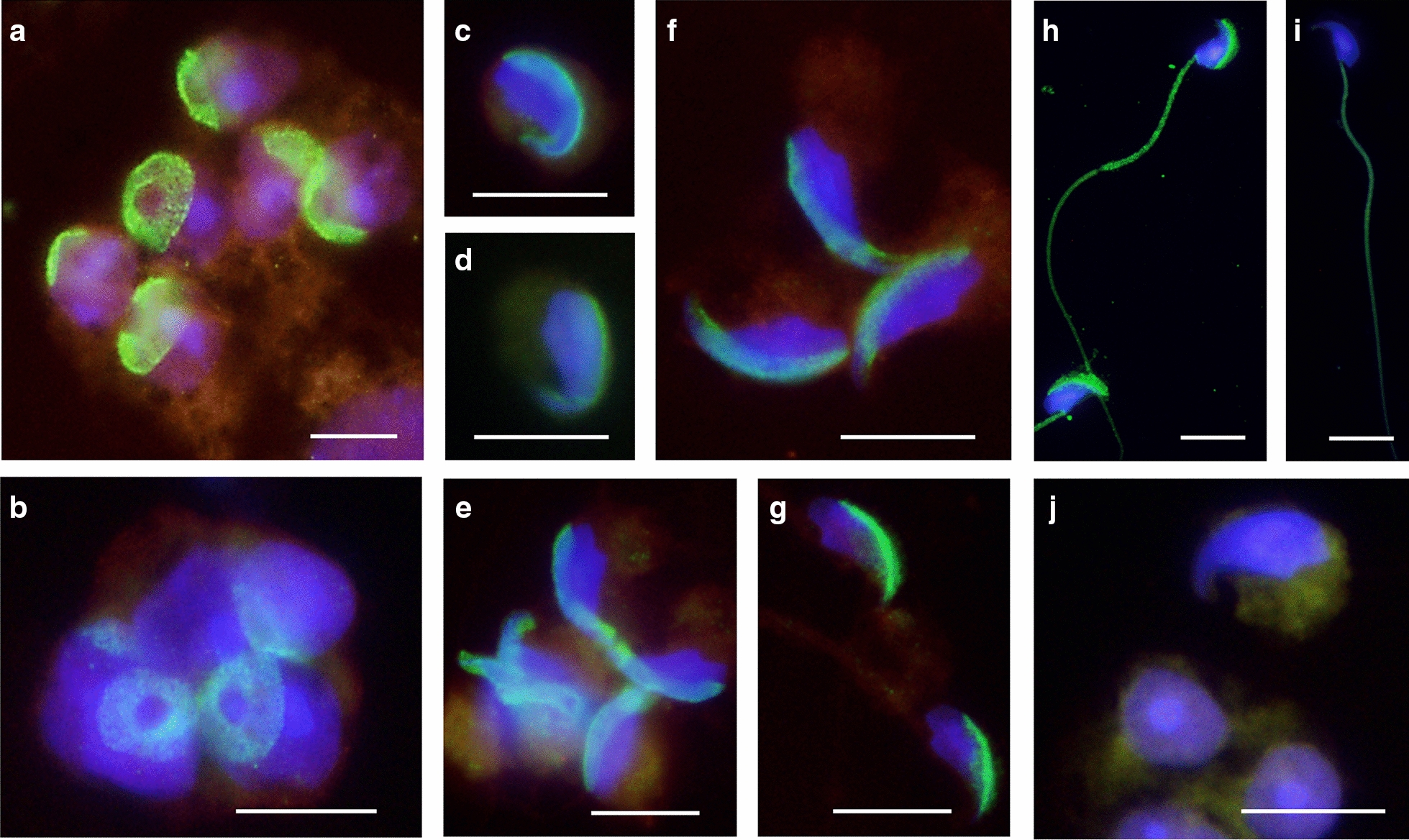
Localization of the mouse PRAMEL1 protein in developing spermatids and spermatozoa. Smash preparations of seminiferous tubules (**A**–**G** and **J**) were stained with anti-PRAMEL1 (green), anti-β-TUBULIN (red) antibody and DAPI (blue). Spermatozoa (**H** and **I**) were stained with anti-PRAMEL1 (green) and DAPI (blue). Merged images for the triple- staining in round spermatids (**A** and **B**), elongating spermatids (**C**–**F**) and elongated spermatids (**G**) were shown. PRAMEL1 staining was observed in acrosomal vesicle region of all the developing spermatids and β-TUBULIN labeling was observed in manchette of elongating and elongated spermatids. PRAMEL1 staining was observed in acrosome and midpiece of sperm tail in mature spermatozoa (**H**). As controls, spermatozoa (**I**) and smashed tubules (**J**) were stained with secondary antibody (FITC-labeled donkey anti-goat IgG and TRITC-labeled donkey anti-mouse IgG) and DAPI. No PRAMEL1-specific staining was observed in the controls. Magnification is × 1000. Scale bar = 10 µm

The mouse *Pramel1* gene (Gene ID: 83491) encodes a protein of 57 kDa as detected by the anti-PRAMEL1 antibody in Western blot analysis, which is predominantly expressed in the testis [33]. IF staining revealed that the PRAMEL1 protein was present in spermatogenic cells and enriched in the acrosomal vesicle region in developing spermatids during acrosome formation (Fig. 1A–G, green color) and in the acrosome and midpiece region of mature spermatozoa (Fig. 1H). PRAMEL1 was highly enriched in the acrosomal vesicle, but not in the acrosomal granule region in round spermatids (Fig. 1A, B). In the elongating (Fig. 1C, F) or elongated spermatids (Fig. 1G), PRAMEL1 formed a layer of concentrated proteins in the acrosome region along the top edge of the hook shaped head, while the β-TUBULIN protein was observed in manchette of elongating and elongated spermatids (Fig. 1A–G, red color). PRAMEL1 appears to be more enriched during the acrosome formation and the condensation of the sperm head. At the end of spermiogenesis, the PRAMEL1 protein was localized primarily in the acrosome and the midpiece of sperm tail where mitochondria sheath is in the mature spermatozoa (Fig. 1H). However, due to the limitation of low-resolution in immunofluorescence staining, it was unclear whether the PRAMEL1 labeling was inside and/or outside of the acrosome in spermatids and spermatozoa.

### Subcellular localization of the mouse PRAMEL1 protein in spermatogenic cells

**Fig. 2:**
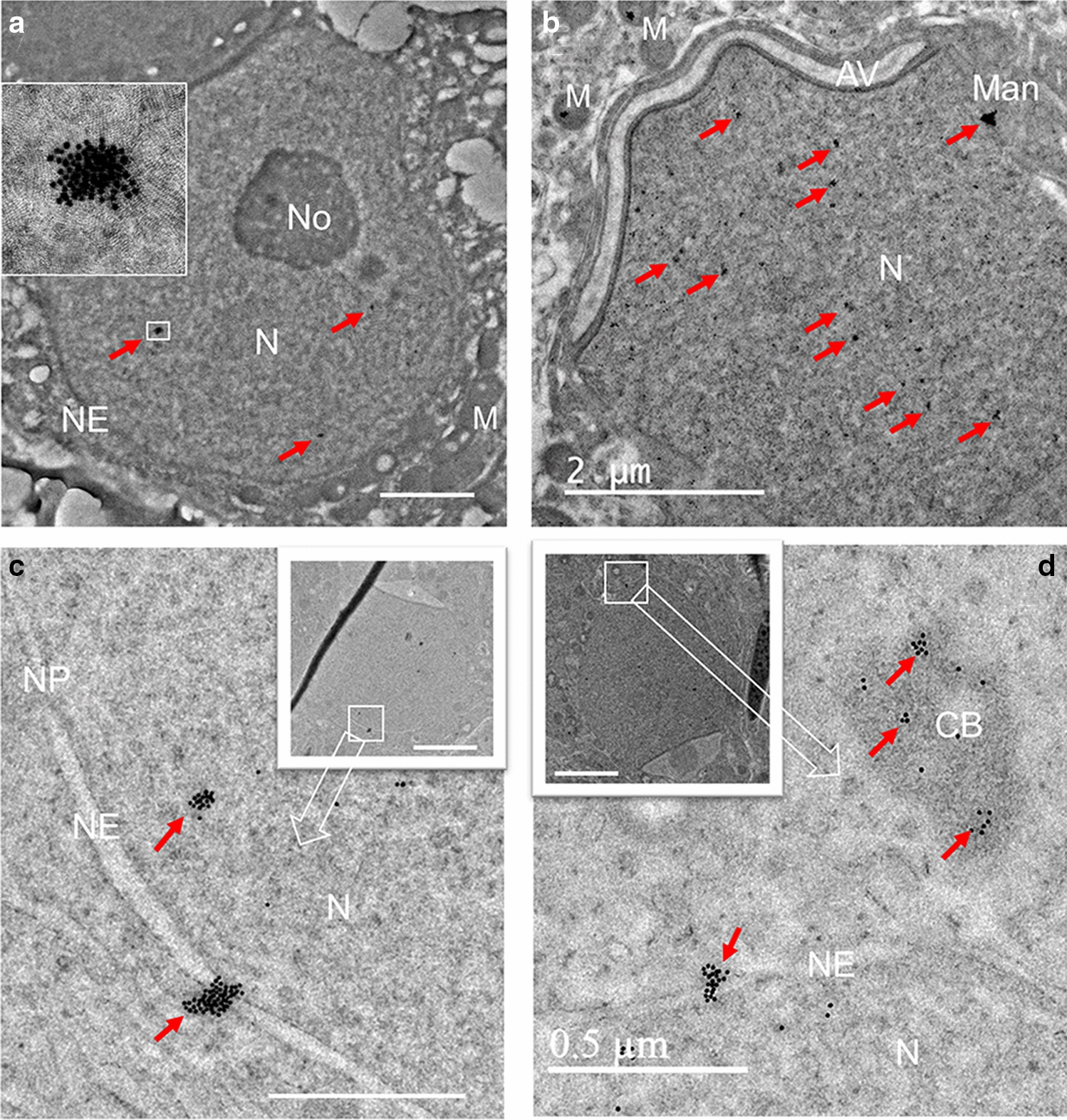
Localization of PRAMEL1 in the nucleus and nuclear envelop of spermatogenic cells. Large clusters of PRAMEL1 gold particle (red arrows) were detected in the nucleus (N) of all types of spermatogenic cells. Low density of clusters was seen in the nucleus of spermatogonia (**A**) and higher density of clusters were seen in spermatids (step 7) (**B**). No PRAMEL1 labeling was seen in the nucleolus (No) of spermatogonia (**A**). Heavy labeling of PRAMEL1 gold particles was detected in the nuclear envelop (NE) or nuclear pore (NP) region in steps 6–7 spermatids (**C**, **D**). AV: acrosome vesicle; CB: chromatoid body; M: mitochondria; Man: Manchette. Scale bar = 2 μm in **A** and **B**, or 0.5 μm (or 2 μm in the insert images) in **C**, **D**

**Fig. 3:**
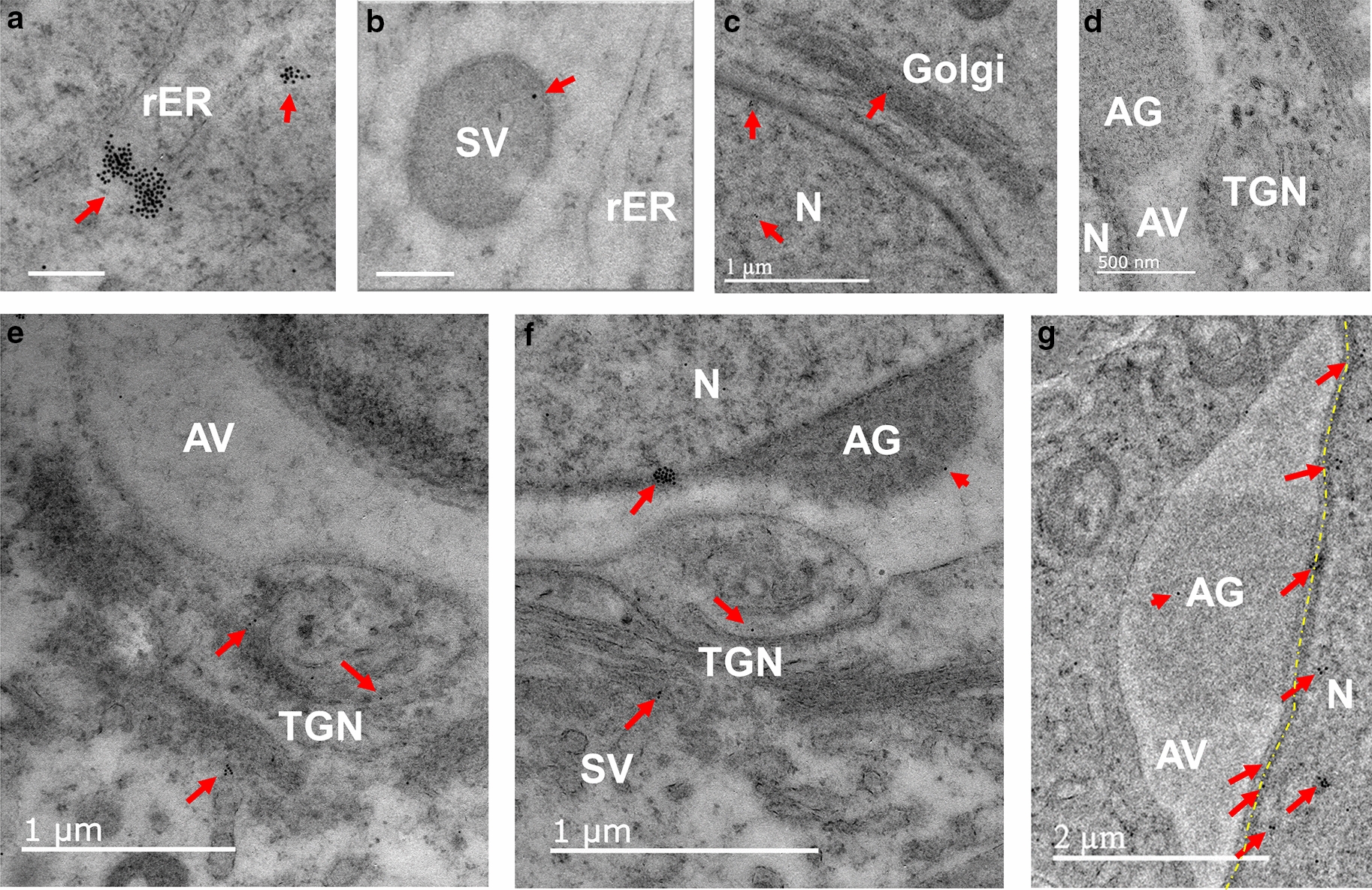
Localization of PRAMEL1 in endoplasmic reticulum (ER), trans-Golgi network (TGN) and acroplaxome of spermatogenic cells. Heavy labeling of PRAMEL1 gold particles was observed in clusters in the rough ER (rER) (**A**), and light labeling was seen in some vesicles (**B**) in the cytoplasm of a spermatocyte. A low density of gold particles was detected in the Golgi apparatus (**C**), but not in acrosome granule (AG) and vesicle (AV) regions in early round spermatids (**D**, **E**). Along the development of acrosome and the formation of the TGN, a low density of PRAMEL1 labeling was seen in TGN of steps 3–4 round spermatids (**E**, **F**). Occasionally, a very few gold particles (arrowheads) were seen in the AG of steps 3–4 round spermatids (**F**, **G**). In contrast, a high density of PRAMEL1 labeling was seen in the acroplaxome region (yellow dotted line) at or cross the nuclear membrane of steps 4–6 spermatids (**F**, **G**). Unlabeled scale bar = 0.2 µm

**Fig. 4:**
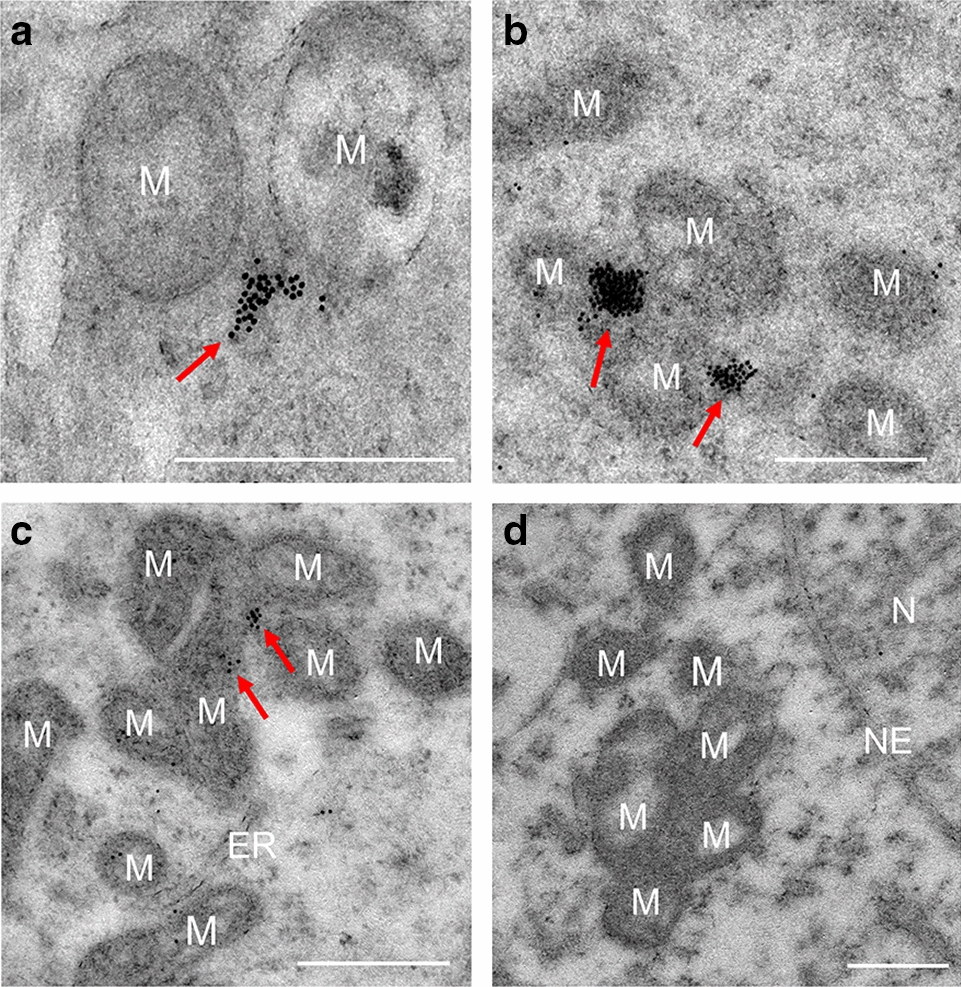
Localization of PRAMEL1 in the intermitochondrial cement (IMC). Mitochondria (M) began to aggregate around the electron-dense material to form IMC in pachytene spermatocytes. Typical IMC with clustered labeling of PRAMEL1 in the cement part of IMC was observed in the early pachytene spermatocyte (**A**), and late pachytene spermatocyte (**B**, **C**). No gold particles were detected in the negative control (**D**). ER: endoplasmic reticulum; N: nucleus; NE: Nuclear envelope. Scale bar = 0.5 µm

**Fig. 5:**
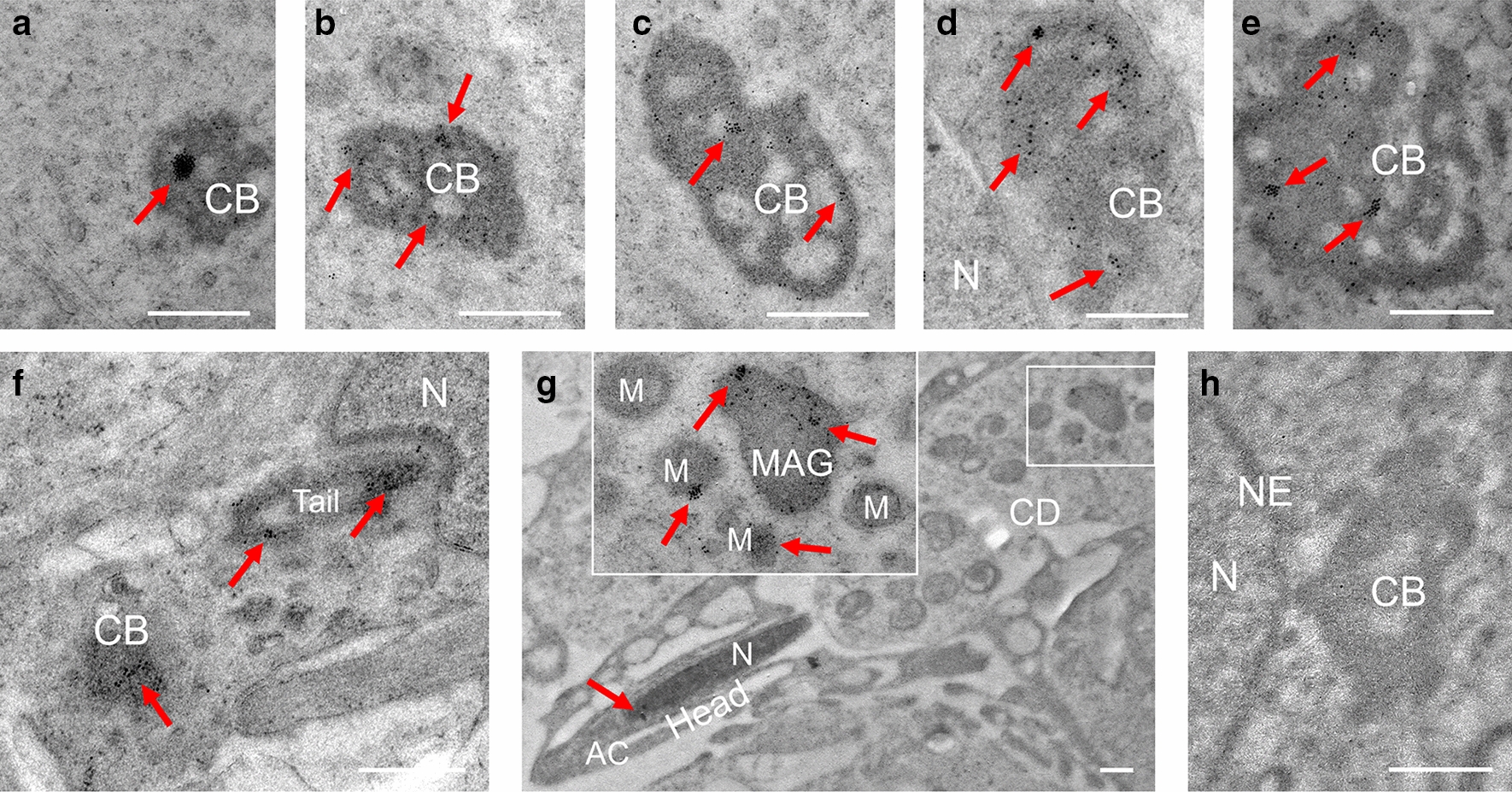
Localization of PRAMEL1 in CB of spermatids. Large clusters of PRAMEL1 gold particles were detected in the early spermatid (step 1) when CB was formed (**A**). As CB continues growing in step 3–4 spermatids (**B**–**E**), the PRAMEL1 protein was enriched and spread over the entire CB. The size of CB decreases when the spermatid begins to elongate (step 5). The image in **F** showed a step 9 spermatid, in which PRAMEL1 particles were clearly seen in the nascent flagellum and the nearby CB. Mitochondria-associated granule (MAG) or late CB was present in the cytoplasm droplet (CD) of elongated spermatids (step14) (**G**). The enlarged image in **G** indicated the enriched PRAMEL1 labeling in the late CB and the associated mitochondria. The gold particles were hardly detected in the nucleus and CB in the negative control (H). M: mitochondria; N: nucleus; NE: Nuclear envelope. Scale bar = 0.5 µm

**Fig. 6:**
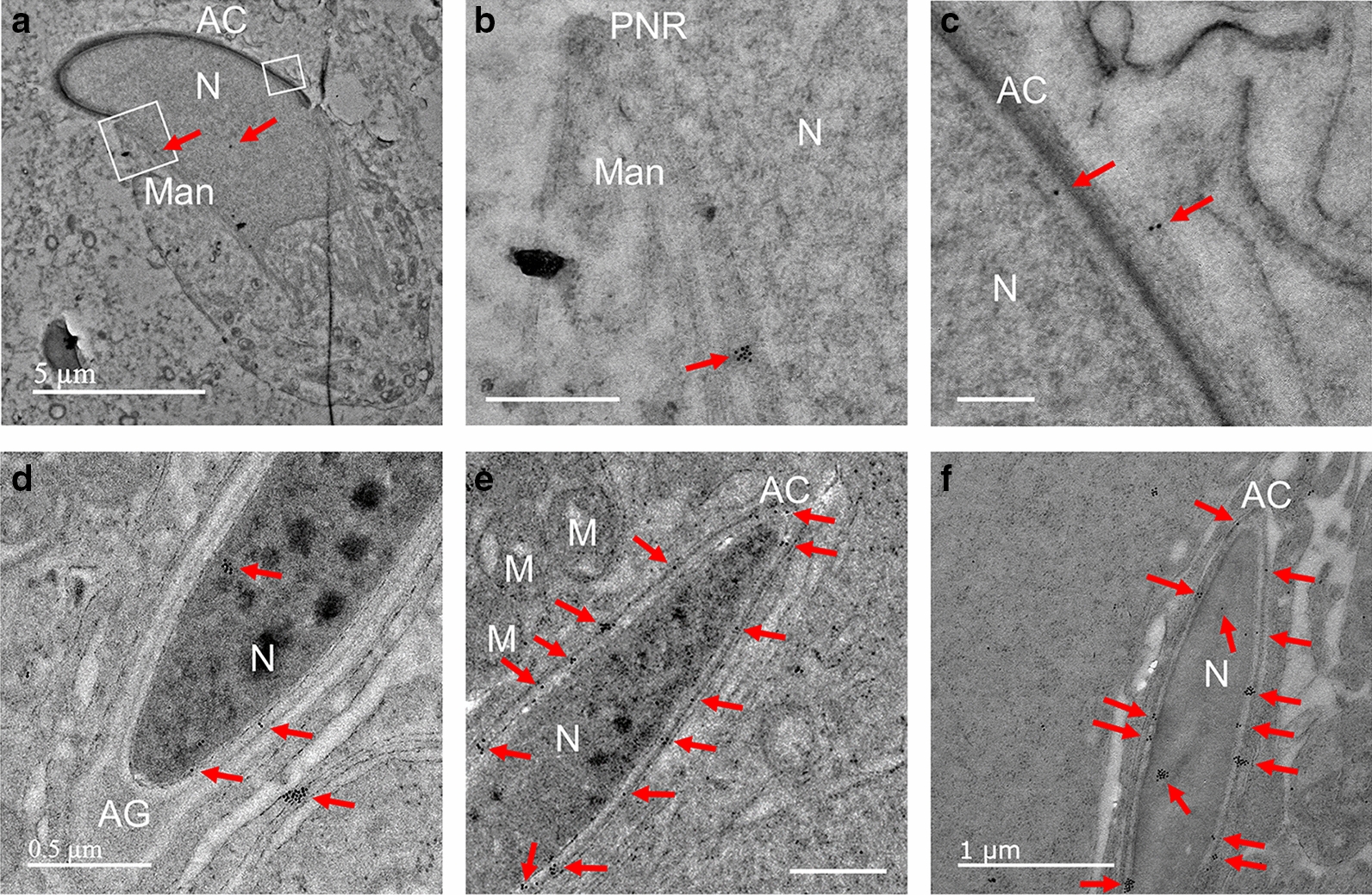
Localization of PRAMEL1 in the acrosome (AC) and manchette (Man). The image in **A** shows a step 9 spermatid. Clusters of PRAMEL1 gold particles were detected in the manchette (boxed region in **A**) and the enlarged image in **B**. A low density PRAMEL1 labeling was seen in the acroplaxome (**C**) (the boxed region in **A**). In steps 10–12 spermatids, clusters of gold particles were seen in the nucleus (N) and acrosome region (**D**, **E**), but a very few gold particles were observed in the acrosome granule (AG) or acrosome matrix (AM) located in the front head (**D**, **E**). In step 16 elongated spermatid, large clusters of gold particles were observed in nucleus and acrosome (**F**). Some of the clusters were in the nuclear membrane adjacent to acrosome. M: mitochondria; PNR: perinuclear ring; Unlabeled scale bar = 0.5 µm

**Fig. 7:**
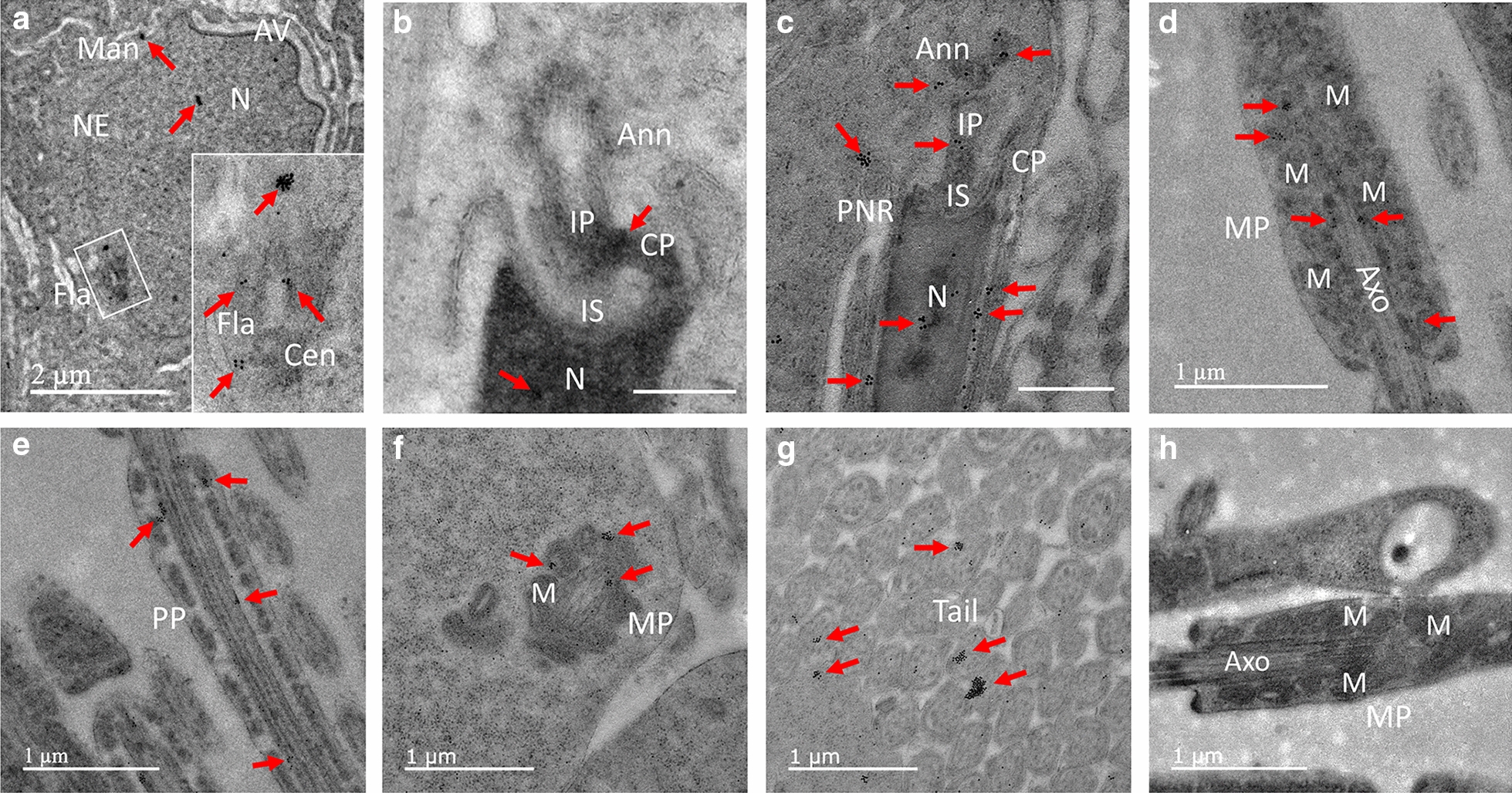
Localization of PRAMEL1 in the flagellum. The initiation of the sperm neck and flagellum (Fla) is seen in the posterior region of the nucleus associated with the proximal and distal centrioles (Cen) in step 7 spermatid (**A**). The inset image enlarges the boxed region (**A**). Heavy PRAMEL1 labeling was observed in clusters in the nucleus (N), manchette (Man), and flagellum (Fla) (A). Immunogold particles were detected in the implantation socket (IS) and plate (IP) of the connecting piece (CP) and the annulus (Ann) during the early development of connecting piece (CP) and midpiece (MP) in steps 8–10 spermatids (**B**, **C**). Large clusters of gold particles were seen in the mitochondrial (M) sheath (**D**, **F**) of MP, and the axoneme (Axo) of the principal piece (PP) of elongated spermatids (**E**). Cross sections of the sperm tail further indicated the localization of PRAMEL1 in flagella (**G**). No specific-labeling was observed in the MP of the sperm tail in the negative control (H). Unlabeled bar = 0.5 µm

IEM was applied to examine the subcellular localization of PRAMEL1 during spermatogenesis. In this technique, the localization of target proteins is determined by gold nanoparticles conjugated to the secondary antibody, which binds to the primary antibody that is directly attached to the target antigen. In general, the localization of PRAMEL1 was clearly seen in different development stages of germ cells, including spermatogonia, spermatocytes, all maturation steps of spermatids and mature spermatozoa (Figs. 2, 3, 4, 5, 6, 7). Large clusters of immunogold particles were especially enriched in the nucleus and the electron-dense materials within the cytoplasm (Figs. 2, 3, 4, 5, 6, 7). To determine whether the large cluster of PRAMEL1 gold particles was an artifact of the secondary antibody aggregation, the secondary antibody was pre-centrifuged before applying to IEM (see Methods). Since no difference in labeling patterns between the centrifuged and non- centrifuged, or between the supernatant and the bottom of the centrifuged secondary antibodies was observed, we concluded that the large clusters of gold particles were not caused by the secondary antibody aggregation. The data presented below were the general descriptions of PRAMEL1 localization patterns in cellular organelles of germ cells.

#### Nucleus

Clusters of PRAMEL1 gold particles were seen clearly in the electron-dense materials of the nucleus in all types of spermatogenic cells, including spermatogonia, spermatocytes and all steps of spermatids (Figs. 2A–C, 3G, 6A, D–F, 7A). The density of the PRAMEL1-labeled clusters in the nucleus varied along with the development of spermatogenic cells. The lowest density was seen in spermatogonia (Fig. 2A), while the highest density was observed in early-mid spermatids (Fig. 2B). In the late spermatids, the density was reduced (Fig. 6D, E), but still higher than that of spermatogonia (Fig. 2A). Interestingly, we frequently observed clusters of heavily labeled gold particles crossing the nuclear envelope (NE) in developing spermatids (Figs. 2D, C, 3F, G, 6F), indicating that PRAMEL1 and its associated proteins are moving across the nuclear membrane. The frequency of gold particle clusters observed in NE was higher in the region where the acrosomal vesicle is attached (Fig. 3F, G). Some of the gold particles were clearly located (or passed through) the nuclear pore (Fig. 2C). There were no gold particles observed in the nucleus and NE of all germ cells in the negative control (Figs. 4D, 5H).

#### Endoplasmic reticulum (ER), small vesicles (SV) and Golgi apparatus

In addition to the nucleus, clusters of PRAMEL1 heavily labeled gold particles were also seen in the rough ER of spermatogonia and spermatocytes (Fig. 3A). There were many small vesicles (SV) of variable sizes present in the cytoplasm of these cells. Some of them contained relatively light electron-dense materials in which a low-density gold particle was observed (Fig. 3B). During the Golgi phase in spermiogenesis, a low density of PRAMEL1 labeling was observed in the Golgi apparatus (Fig. 3C, D). A single gold particle or small clusters were seen in the trans-Golgi network (TGN) in the cap (and acrosome) phase of round spermatids (Fig. 3E, F). In general, there was no PRAMEL1 labeling observed in the acrosomal vesicle (AV) (Fig. 3D–G), and a very few gold particles were detected in the acrosome granule (AG) region (Fig. 3D–G). The IEM data was not completely in line with the IF staining results (Fig. 1A, B). Remarkably, large clusters of gold particles were frequently seen in the acroplaxome, a microtubule structure at the boundary of acrosome and the nucleus (Fig. 3F, G). At a cellular level, this high density of PRAMEL1 gold particles around the acroplaxome could form a condensed layer, which explains why a ring-shape of fluorescent signals was seen in the AV region in the cap phase round spermatids (Fig. 1A, B).

#### Intermitochondrial cement (IMC)

IMCs are electron-dense materials found between mitochondria in the cytoplasm of early and late spermatocytes, especially in the pachytene spermatocyte (Fig. 4). The number of mitochondria aggregated in IMCs varied dramatically from two (Fig. 4A) to a dozen (Fig. 4C). The IEM staining results indicated that the PRAMEL1 immunogold particles were seen in huge clusters in the cement portion (electron-dense region) of IMCs, but not in the mitochondria itself (Fig. 4A–C). The PRAMEL1 labeling was observed in the early pachytene spermatocyte (Fig. 4A) when the IMCs begin to form, to the late pachytene spermatocyte (Fig. 4C). These mitochondria-associated gold particles were also seen in the cytoplasm of developing spermatids [see mitochondria-associated granule (MAG) in Fig. 5G] and in the mitochondrial sheath within the midpiece of sperm tail (Fig. 7D, F). Therefore, PRAMEL1 protein is present not only in IMCs, but also in the electron-dense materials that are associated with mitochondria in different compartments of spermatogenic cells at the different development stages, signifying a potential role of PRAMEL1 related to the function of mitochondria in germ cells.

#### Chromatoid body (CB)

The mouse CB has a typical honey-comb structure with cloud-like non-membrane electron-dense materials (Figs. 2D; 5A–E). The CB was first observed in early round spermatids (Fig. 5A). The size of CB increased as round spermatids progressed through development (steps 1–5) (Figs. 2D, 5A–E), however the size significantly decreased during the elongation of spermatids (Fig. 5F). The location of CB in the cytoplasm changed from areas close to the nucleus (Figs. 2D, 5D) to the distal region of the nucleus where the flagellum formed (Figs. 2D, 5F). In late steps of spermatids, the CB material was often associated with mitochondria, and termed as mitochondria-associated granule (MAG) (also known as the late CB). MAG or late CB was present in the cytoplasmic droplet (CD) in elongated spermatids (Fig. 5G) and disappeared in the mature spermatozoa. Intensive labeling of PRAMEL1 was observed in clusters in CB of early spermatids (Fig. 5A). In mid-spermatids, the gold particles tend to be spread over the entire CB in smaller clusters (Fig. 5B–E). During elongation, the size of CB decreased as the honey-comb structure disappeared, and the PRAMEL1 labelling was consistently observed in the electron-dense materials of CB until the very late steps (Fig. 5G), where the late CB or MAG was present in the CD region (Fig. 5G).

#### Acrosome and manchette

In contrast to the strong PRAMEL1 fluorescent staining in the acrosome region of elongated spermatids (Fig. 1C–G), the PRAMEL1 immunogold labeling hardly detected any gold particle in the AV and AG regions of steps 1–7 spermatids (Figs. 3C–G, 6A–C). In early elongating spermatids, a few gold particles were seen in the acrosomal region (Fig. 6A, C). However, during development of the acrosome throughout spermatid elongation, an increased density of gold particles was observed in the membrane either at the nuclear-inner acrosome membrane (IAM) junction or the outer acrosome membrane (OAM) (Fig. 6E, F), but not in the AG or acrosome matrix (AM) region (Fig. 6D). We observed large clusters of gold particles within the longitudinal section of manchette (Figs. 2B, 6A, B), which is a sheath of microtubules that starts at the perinuclear ring (PNR), surrounds, and extends tailward from the nucleus of elongating spermatids (Fig. 6A, B). The location of the PRAMEL1 labeling in these microtubules varied from cell to cell based on their developmental stage, suggesting that the PRAMEL1-associated protein complex may move along manchette microtubules. However, no labeling was detected in the PNR region (Fig. 6B).

#### Centrioles, annulus, and flagellum

Clusters of PRAMEL1 labeling were observed essentially in the microtubules of both the proximal and the distal centrioles that had attached to the nuclear envelope (Fig. 7A). A low density of immunogold particles was seen in the newly formed implantation socket (IS) and plate (IP) of the connecting piece (CP) (Figs. 5F; 7B, C). Annulus was visible in steps 7–8 spermatids and moderate labeling was seen in the annulus structure. Intense labeling was seen in clusters in the outer dense fibers surrounding the axoneme in the midpiece (Fig. 7D) and principal piece (Fig. 7E) of the flagellum based on images from lateral and longitudinal sections. However, the cross-section images showed that the heavily labeled clusters were often associated with the electron-dense material between the mitochondria in the midpiece and much less labeling was seen in the microtubules of the axoneme in mid and principal pieces of the sperm flagellum (Fig. 7F, G). Similar to the observation in manchette, the location of the PRAMEL1 labeling in the flagellum varied from section to section based on their developmental stage, suggesting the movement of PRAMEL1 clusters along microtubules in the sperm tail.

### Subcellular localization of the mouse PRAMEX1 protein

**Fig. 8:**
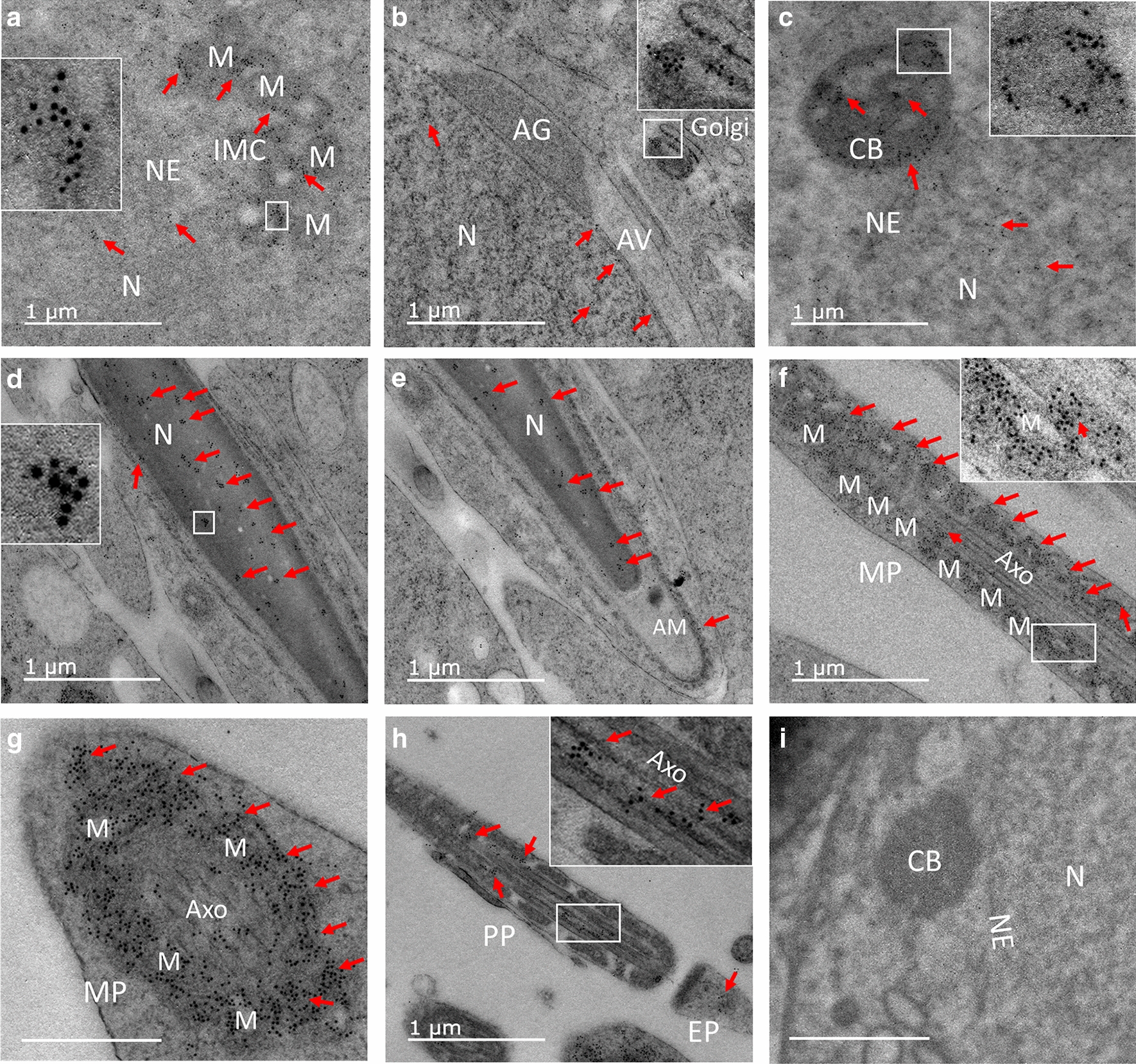
Subcellular localization of the mouse PRAMEX1 in spermatogenic cells by IEM. Heavy PRAMEX1 labeling (red arrows) was observed in the cement region of intermitochondrial cement (IMC) in spermatocytes (**A**). A low density of gold particles was seen in the nucleus (N) of spermatocytes (A) and round spermatids (**B**, **C**). In contrast, a high density of clustered labeling was seen in elongated spermatids (**D**, **E**). No labeling was observed in the acrosome granule (AG) and acrosome vesicle (AV) (**B**) and acrosome matrix (AM) (**E**), though a low to moderate density of PRAMEX1 labeling was clearly seen in the Golgi apparatus and acroplaxome (**B**). The heaviest labeling of PRAMEX1 was seen in the electron-dense materials surround mitochondria (M) of midpiece (MP) in lateral (**F**) and cross sections (**G**). Clusters of gold particles were also seen in the microtubules of axoneme (Axo) in the MP (**F**, arrowhead) and principal piece (PP) (**H**) and the end piece (EP) (**H**) of the flagellum. Gold particles were hardly detected in the negative control (**I**). Inserts enlarge the boxed regions (**A**–**D**, **F**, **H**). Scale bar = 1 µm

The localization of PRAMEX1 at a subcellular level was very similar to that of PRAMEL1, as both proteins were detected mainly in the nucleus of spermatogenic cells, IMC of spermatocytes, CB of spermatids and the midpiece and principal piece of the flagellum (Fig. 8). To avoid redundancy in description of all PRAMEX1 IEM data, we focused on the key differences between PRAMEL1 and PRAMEX1 labeling.

Clusters of gold particles were observed in the nucleus of all types of spermatogenic cells, including spermatogonia, spermatocytes and spermatids (Fig. 8A–E). The gold particles of PRAMEX1 labeling were clearly seen in the nucleus with a low density in spermatocytes and round spermatids and a high density in elongated spermatids (Fig. 8D, E). We did not observe any cluster of PRAMEX1 gold particles at or cross the nuclear envelope, even though low density labeling was detected at the acroplaxome (Fig. 8B).

PRAMEX1 labeling was detected in the electron-dense materials surrounding mitochondria in different types of spermatogenic cells including spermatocyte and spermatids, particularly in the IMC (Fig. 8A). In contrast to PRAMEL1 labeling (Fig. 4), PRAMEX1 labeling was highly enriched, and widespread across the entire IMC (Fig. 8A). It was also enriched in CB (Fig. 8C). The pattern of gold particle of PRAMEX1 in CB was very similar to that of PRAMEL1 (Fig. 5A–E). During acrosome formation and the morphological change in the nuclear shape, a low to middle level of PRAMEX1 labeling was detected in the Golgi apparatus and acroplaxome, but not in the AG and AV regions of round spermatids (Fig. 8B) or the AM region of the elongated spermatids (Fig. 8E).

The most significant difference between the PRAMEL1 and PRAMEX1 labeling was found in the flagellum (Figs. 7 and 8). Although both proteins were detected in the midpiece and principal piece of the sperm tail, PRAMEX1 labeling was highly enriched in the midpiece where the mitochondrial sheath is located. Like the labeling seen in IMC, the electron-dense materials around mitochondria in this region were completely labeled (Fig. 8F, G). In contrast, limited or no labeling was observed inside mitochondria (Fig. 8A, F, G). Large clusters of PRAMEX1 labeling were seen in outer dense fibers of the axoneme in the midpiece (Fig. 8F, G) and principal piece (H). A low density of PRAMEX1 labeling was seen in the end piece of the sperm tail (Fig. 8H).

### Co-localization of the mouse PRAMEL1-DDX4 and PRAMEL1-KIF17b in germ granules

**Fig. 9:**
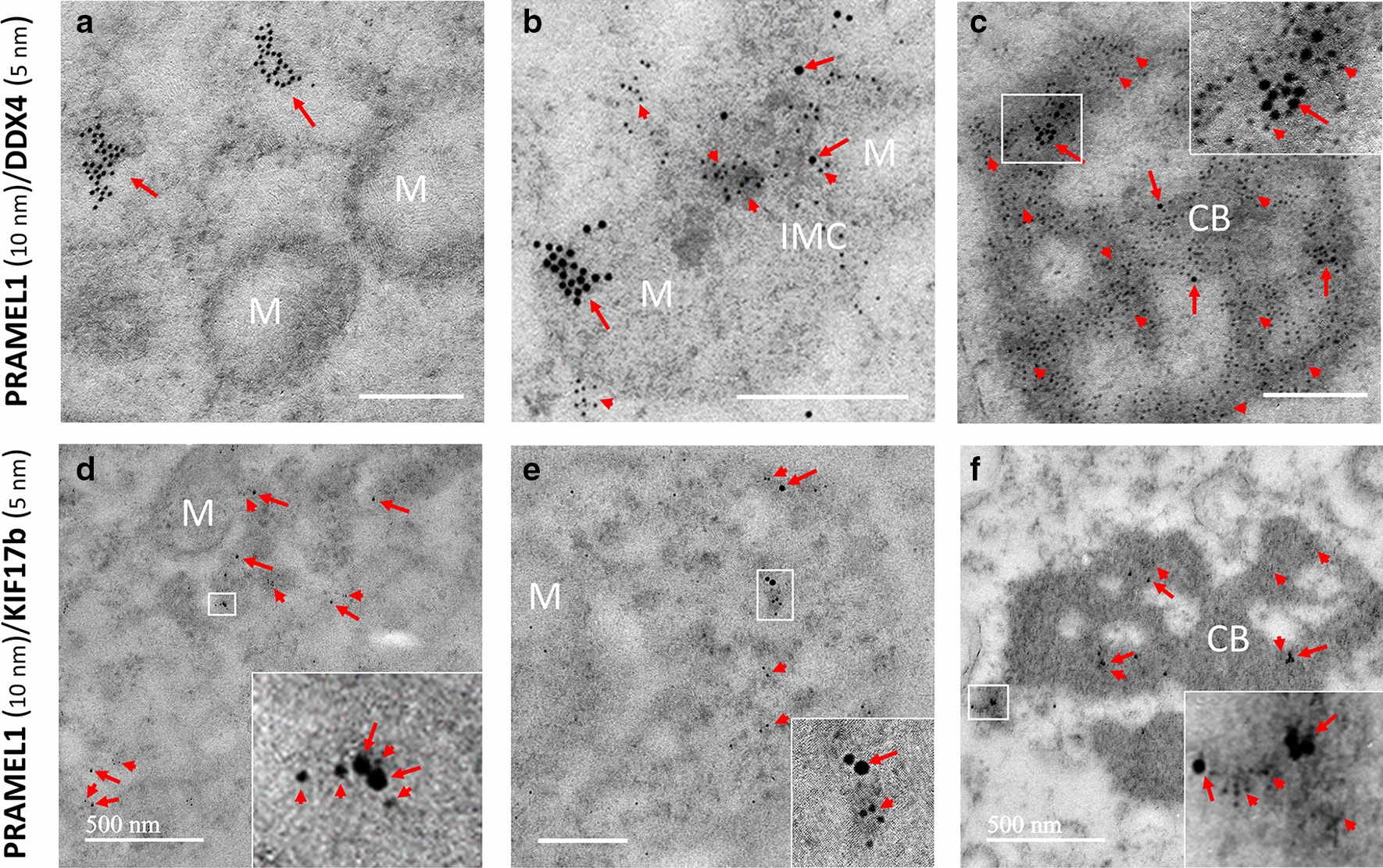
Co-localization of the mouse PRAMEL1 and DDX4 or KIF17b in germ granules of spermatogenic cells by IEM. Both PRAMEL1 (red arrows, 10 nm gold particles) and DDX4 (red arrowheads, 5 nm gold particles) immunogold particles were detected in the IMC (**A**, **B**) and CB region (**C**). The clusters of PRAMEL1 and DDX4 labeling could be overlapped (**C**), but not always correlated with each other (**A**, **B**). In contrast, PRAMEL1 (red arrows, 10 nm gold particles) and KIF17b (red arrowheads, 5 nm gold particles) labeling was colocalized in the same region of the IMC (**D**, **E**) and CB (**F**). Inserts enlarge the boxed regions (**C**–**F**). Unlabeled bar = 0.25 µm

Both PRAMEL1 and DDX4 proteins were localized in the germ granules of spermatogenic cells (Fig. 9A–C). Before the formation of IMC in spermatocytes, a heavy labeling of PRAMEL1 gold particles was seen in cytoplasm close to mitochondria, but very limited DDX4 labeling was seen in the neighborhood (Fig. 9A). In late spermatocytes when the IMC formed, large clusters of both PRAMEL1 and DDX4 labeling were observed in the cement portion of IMC (Fig. 9B). As PRAMEL1 and DDX4 labeling was overlapped in one region and separated in another region of IMC (Fig. 9B), we concluded that these two proteins were not correlated to each other. As shown in Fig. 5, large clusters of PRAMEL1 labeling were seen in CB of round spermatids, which was confirmed in the co-localization experiment (Fig. 9C). Compared to PRAMEL1 labeling, DDX4 had a dramatic increase in the density of immunogold particles that evenly distributed across the entire electron-dense region in CB (Fig. 9C). Interestingly, DDX4 labeling was hardly detected in the nucleus where heavy PRAMEL1 labeling was observed (Fig. 9C), indicating that PRAMEL1 and DDX4 proteins would not be located in the same protein complex.

Previous studies found that the mouse KIF17b was present in the nucleus and CB [41, 42]. Dual-staining with PRAMEL1 and KIF17b antibodies in our co-localization IEM analysis indicated that both proteins were detected in the electron-dense material associated with mitochondria, IMC (Fig. 9D, E), and CB (Fig. 9F). The gold particles from both PRAMEL1 and KIF17b labeling tended to be close to each other or overlapped in IMC and CB (Fig. 9D–F), implying that PRAMEL1 and KIF17b labeling were correlated or complexed together in germ granules.

## Discussion

Recent studies on the *PRAME* gene family have revealed that different members of the PRAME family are involved in germ line development and gametogenesis at different developmental stages (for a review, see [39]). For instance, the mouse *Gm12794c* and *Pramel7* are essential factors in the signaling network regulating pluripotency and self-renewal of embryonic stem cells (ESCs) [36–38], while the mouse *Pramef12* is crucial for spermatogonia and spermatogonia stem cell (SSC) development [35]. Deletion of *Pramef12* leads to smaller testes with a Sertoli cell-only (SCO) phenotype in mature mice that are infertile [35]. Our previous work demonstrated that both the mouse *Pramel1* and *Pramex1* are predominantly expressed in the testis and involved in spermatogenesis [33]. A recent study on the conditional *Pramex1* KO (cKO) mice revealed that PRAMEX1 functions in germ cell development, especially during the first round of spermatogenesis [34]. It is evident that proteins encoded by members of the *PRAME* gene family act as nuclear transcriptional factors that are essential for germ cell development. However, what function(s) PRAME family proteins may have in the cytoplasm of germ cells is still unknown. A physical localization of the PRAME proteins on cellular organelles would help us to understand the functional role of these proteins during spermatogenesis. To date, the only member in the mammalian PRAME family that has been characterized at a subcellular level is the bovine PRAMEY, which is highly enriched in organelles such as the nucleus, IMC, CB, AG, acrosome and flagellum in spermatogenic cells [19]. In the present study, we performed a systematic IEM analysis on the mouse PRAMEL1 and PRAMEX1 proteins. We found that the protein localization patterns of the mouse PRAMEL1 were very similar to that of the bovine PRAMEY, except for the AG and AM regions where the PRAMEY protein was highly enriched in the bovine spermatids, but the mouse PRAMEL1 (or PRAMEX1) was hardly detected in these regions. Instead, both the mouse PRAMEL1 and PRAMEX1 proteins were enriched in the acroplaxome at a subcellular level, very similar to the distribution pattern of the testis FER (Fer tyrosine kinase) in acroplaxome [43]. FER regulates cell–cell adhesion and mediates signaling from the cell surface to the cytoskeleton via growth factor receptors [43]. Without the IEM data, PRAMEL1 was misinterpreted as an acrosome-located protein based on the immunofluorescent staining in the present (Fig. 1) and previous report [33], demonstrating the need for doing a high-resolution protein localization at a subcellular level.

In IEM analysis, the detection and localization of a specific protein depends upon the antigen-recognition specificity of the primary antibodies [44]. In this study, the anti-PRAMEL1 antibody was raised against the mouse PRAMEL1 peptide sequence, but the anti-PRAME antibody was against human (h) PRAME. Although the peptide used to make the hPRAME antibody has a 92% amino acid sequence similarity to the mouse PRAMEX1 protein, the immunoreaction (gold labeling) we observed in the mouse germ cells may not be specific to the mouse PRAMEX1. Given the multiple copies of the *Prame* gene family in the mouse genome, the anti-hPRAME antibody used in this work could potentially interact with other proteins in the mouse *Prame* gene family. Therefore, detailed analysis of all proteins in the *Prame* gene family is needed if gene-specific antibody is available for all members in the *Prame* gene family. Despite this limitation, our IEM data with the hPRAME antibody still provided us with invaluable information about the localization of the PRAMEX1 (previously known as PRAME) proteins in spermatogenic cells.

**Fig. 10:**
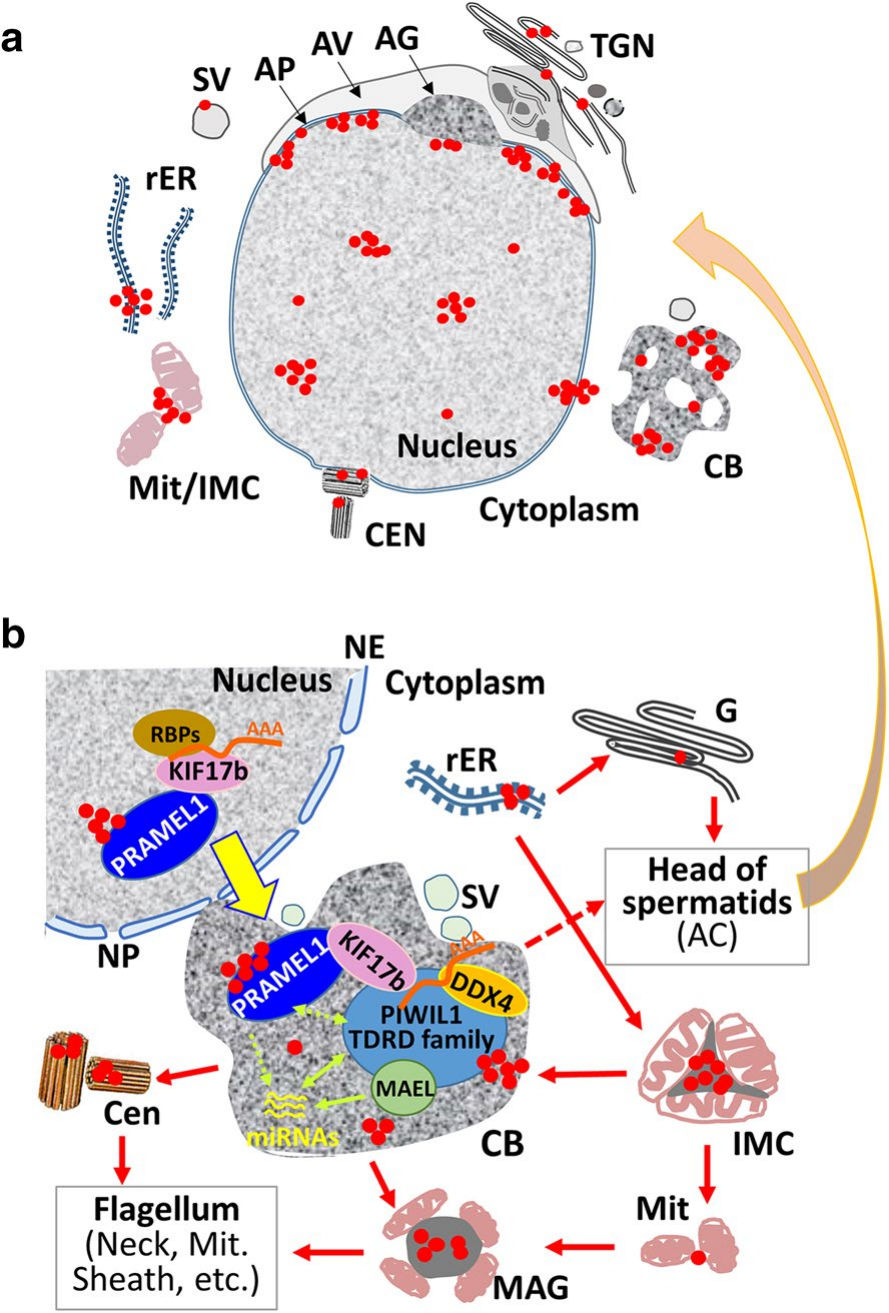
Schematic representation of PRAMEL1 localization in germ cells and a hypothetical model for PRAMEL1 in protein trafficking during spermiogenesis. **A** Schematic representation of PRAMEL1 localization in a round spermatid as revealed by IEM. PRAMEL1 proteins are enriched in the nucleus (N), intermitochondrial cement (IMC), chromatoid body (CB) and the acroplaxome (AP) region in round spermatids. **B** A hypothetical model for PRAMEL1 action in the “Nucleus-CB-organelle” transport system, modified from references [41, 65]. In early round spermatids, the newly formed CB becomes the center for RNA metabolism and small RNA-mediated regulation of gene expression [6, 7]. CB makes frequent contacts with the nuclear envelope (NE), Golgi apparatus (G), mitochondria (Mit), centrioles (Cen), and other cellular organelles, to ensure material continuities. The protein complex that contains MIWI, MAEL, DDX4, and the TDRD family are primarily located in CB. The RNA binding proteins (RBPs) bind to the RNA which forms a complex with the kinesin motor protein KIF17b. KIF17b shuttles between nuclear and cytoplasmic compartments and interacts with PIWIL1-TDRD1-MAEL protein complex in CB. PRAMEL1 is likely to play a role in the KIF17b-mediate transport. Red dots represent the PRAMEL1-labeled immunogold particles. Solid arrows indicate known interactions between organelles, while dashed arrows show potential interactions. AC: acrosome; AG: acrosome granule; AV: acrosome vesicle; Cen: centrioles; Mit: mitochondria; NP: nuclear pore: rER: rough ER; SV: small vesicles; TGN: trans-Golgi network

Our IEM data indicated that the PRAMEL1 and PRAMEX1 proteins shared a similar subcellular localization pattern in spermatogenic cells. They were both primarily localized in the nucleus, germ granules (IMC and CB), and flagellum (summarized in Fig. 10A). All these organelles are crucial for spermatogenesis [45]. Apart from the similarities between the localization patterns of these two proteins, there were also significant differences between the localization patterns of the two proteins: the PRAMEL1 protein complex was frequently found to be in the nuclear membrane or nuclear pore region, whereas PRAMEX1 was not found. Another difference was that PRAMEX1 was extensively enriched in the electron-dense materials associated with mitochondria, such as IMC of spermatocytes and the mitochondrial sheath in the midpiece of elongated spermatids. In comparison, the PRAMEL1 protein was in clusters in these regions, but not as widely spread as the PRAMEX1 protein. The overlapped, but not identical protein localization patterns between PRAMEL1 and PRAMEX1 suggest that different members of the PRAME family may work redundantly or complementarily when functioning collectively in germ cells. This is supported by a recent report on PRAMEX1 cKO mice [34]. Even though the PRAMEX1 cKO (mature) males have a significantly smaller testis, an evident increase in germ cell loss and decrease in sperm count, they are still fertile. Complementary gene function from other members of the *Prame* gene family was considered as the reason for normal fertility of the PRAMEX1 mutants [34].

As a nuclear receptor transcriptional regulator, the hPRAME functions in transcription regulation in cancer cells by suppressing RA signaling through its interaction with retinoic acid receptors (RARs) [25, 46]. Binding of RA induces a change in the conformation of the RAR ligand binding domain (LBD), promoting the recruitment of co-activator complexes with histone acetyltransferase activities. These changes decrease transcription of RA target genes, regulating differentiation, cell cycle arrest and apoptosis pathways in responsive cells [25, 46]. The hPRAME also binds to histone H3 and occupies a transcriptionally active promoter region that is often bound by nuclear transcription factor Y (NFY) [47, 48]. Data from the cancer biology research suggest that PRAME’s role in transcriptional regulation could be multifaceted. In this work, we found that the density of the mouse PRAMEL1 protein complexes in the nucleus increased from spermatogonia to spermatids along the development of spermatogenic cells. The highest density was seen in round spermatids before elongation (summarized in Fig. 10A). We speculate that the degree of density in immunogold particles could be an indicator for the spatial–temporal activity of PRAMEL1 in transcriptional regulation during germ cell development.

In addition to the nucleus, we found that both PRAMEL1 and PRAMEX1 proteins are enriched in several important cytoplasmic organelles of spermatogenic cells (Fig. 10A, B), suggesting that their function is likely beyond the regulation of transcription. Based upon the unique horseshoe-shape structure of the LRR domains in the PRAME family and the wide distribution of PRAMEL1 and PRAMEX1 during spermatogenesis, we propose that the PRAMEL1/PRAMEX1 proteins may function in protein trafficking through different mechanisms. One possible mechanism is the ‘ER-Golgi-acrosome’ biosynthetic transport system as described in the previous report on the bovine PRAMEY [19], where proteins of the PRAME family play an important role in acrosome biogenesis. In fact, studies in the human cancer cells also revealed that PRAME is associated with Cullin E3 ubiquitin ligase complexes in the Golgi and plays a role in ubiquitylation of target proteins [49–51].

Another possible mechanism that PRAMEL1/PRAMEX1 is involved in could be the microtubule-based transport system in germ cells. Microtubules are the prime component of the cytoskeleton along with microfilaments, and the basic structure of flagellum and manchette, which provide the highway for transport in late spermatids [52, 53]. Thus, the microtubule-based transport is essential for spermatogenesis. Microtubular dysfunctions have been found to be associated with male infertility (for a review, see [54]). The kinesin superfamily proteins (KIFs) are microtubule-based molecular motors that convert the chemical energy of ATP hydrolysis to the mechanical force of transporting various types of cargo, including vesicles, proteins or RNAs along the microtubules [55]. Several KIFs, including KIFC1 and KIF17b, have been reported to play a role in spermatogenesis. KIFC1 is associated with the acrosome and manchette in developing spermatids [56–58], and plays a role in vesicle trafficking from Golgi to acrosome during acrosomogenesis [19, 58, 59]. In the manchette, KIFC1 serves a structural role to transport molecules along the microtubules by interacting with a LRR protein PPP1R42 (protein phosphatase 1 regulatory subunit 42) [58, 60]. In this work, we demonstrated that two new LRR proteins, PRAMEL1 and PRAMEX1 [39], are enriched in the microtubules of the acroplaxome, manchette, and axoneme. The dynamic localization of PRAMEL1 and PRAMEX1 proteins in these microtubule-based organelles is an indication of their involvement in protein trafficking during spermiogenesis. It remains to be investigated whether the PRAME family proteins interact with KIFC1 or any other KIFs in the microtubule-based transport in germ cells.

The third possible mechanism involves the nucleus, germ granules (IMC and CB) and the development of cellular organelles. We referred it as the “nucleus-CB-organelle” transport system (summarized in Fig. 10B), in which PRAMEL1 and its associated protein complex serve as a shuttle to transport proteins and other molecules necessary to the formation of new organelles and development of germ cells. As a typical germ granule and a characteristic of germline cells, CB is a ribonucleoprotein (RNP) amorphous aggregate without a membrane, and its molecular composition is evolutionarily conserved in divergent species from Drosophila to mammals [61, 62]. Since CB is enriched in mRNAs, miRNAs, and piRNAs, RNA-binding proteins, RNA helicases and other proteins involved in various RNA regulatory pathways, it has been considered as a center for RNA metabolism and small RNA-mediated regulation of gene expression in haploid male germ cells [6, 7]. Therefore, CB is the core in the “nucleus-CB-organelle” system (Fig. 10B). Previous studies indicated that CB is essential during spermatogenesis by coming in contact with other subcellular organelles, such as the nucleus, mitochondria, and Golgi complex [17, 18]. It moves in the cytoplasm and makes connections frequently with the nuclear envelope to ensure material continuities [41, 63]. In addition, it passes through cytoplasmic bridges to the adjacent spermatids, and provides a system to share intercellular materials between spermatids [64]. In this work, our IEM data clearly indicated that the mouse PRAMEL1 and PRAMEX1 proteins are localized in both the nucleus and germ granules, including CB, IMC, and MAG (Fig. 10B). The behavior and dynamic movements of the PRAMEL1- and PRAMEX1-labeled CB were similar to those CBs observed in rats, mice and cattle [8, 17, 19, 63]. Most importantly, our IEM data provided the first direct evidence that the PRAMEL1 associated protein complex is localized at or passed through the nuclear envelope (Fig. 2), signifying a potential role of PRAMEL1 in nucleocytoplasmic transport (Fig. 10B).

As a male germ cell-specific granule, CB contains several key proteins that are essential for spermatogenesis [17, 18]. Among them, PIWIL1 (also known as PIWI, MIWI, or CT80) plays important roles in stem cell self-renewal, RNA silencing, and translational regulation [12, 13, 66]. TDRD1 (also known as CT41) functions in the suppression of transposable elements during spermatogenesis by forming a complex with piRNAs and PIWIL1 proteins to promote methylation and silencing of target sequences [14, 15]. Another germ cell-specific transposon silencer–MAEL (also known as CT128), is also a component of this large protein complex: PIWIL1-TDRD1-MAEL (Fig. 10B) [16]. All these three proteins are cancer-testis (CT) antigens, or CTAs, which are predominantly expressed in germ cells of testes and a variety of tumors. In addition, an RNA helicase, DDX4, is highly enriched in and serves as a biomarker for CB [9, 67]. DDX4 has been shown to regulate transcription, RNA unwinding, and mRNA nuclear export and translation [68, 69]. Although the functional role of PRAMEL1 in germ cells is still poorly understood, the co-existence of PRAMEL1 with DDX4 and other important CTAs in CB indicates the involvement of the PRAME gene family in the CB function during spermiogenesis.

The mobility of CB is based on the intracellular microtubular network in the cytoplasm [64], and a kinesin protein, KIF17b, has been found to be involved in CB movements. KIF17b shuttles between nuclear and cytoplasmic compartments in haploid round spermatids and interacts with PIWIL1 as part of the PIWIL1-TDRD1-MAEL protein complex (Fig. 10B) [41, 42]. To test whether PRAMEL1 is part of the KIF17b protein complex in nucleocytoplasmic transport, co-localization of PRAMEL1 and KIF17b was done in the present study. Our IEM data provided clear evidence that the PRAMEX1 and KIF17b labeling were correlated to each other (Fig. 9D–F). In contrast, the PRAMEL1 and DDX4 labeling patterns were not correlated (Fig. 9A–C), indirectly supporting that PRAMEX1 and KIF17b may function together in CB.

It is worth noting that the presence of the mouse PRAMEL1 and PRAMEX1 proteins observed in this work and of the bovine PRAMEY protein reported in a previous study [19] was always associated with mitochondria during germ cell development. PRAME proteins were first aggregated with mitochondria in spermatocytes during meiosis to establish IMC that transformed into CB in haploid spermatids. Towards the end of spermiogenesis, mitochondria once again were in contact with CB, resulting in the formation of MAG. At the end of spermatogenesis, PRAME proteins were highly enriched in the mitochondrial sheath of flagellum in mature spermatozoa. Given the facts that KIF-mediated microtubule-based transport, CB mobility and the sperm motility are all ATP-dependent activities, it is reasonable to hypothesize that PRAME proteins may be involved in energy transfer in germ cells, though this hypothesis remains to be tested in the future study.

## Conclusion

In the present study, the detailed subcellular localization of the mouse PRAMEL1 and PRAMEX1 proteins in spermatogenic cells was uncovered. The results suggest that the immunogold labeling intensity of these proteins in the nucleus may reflect the gene activity in the nuclear transcription, and the dynamic distribution of these proteins in several important cellular organelles, such as germ granules (IMC and CB), manchette and flagellum, indicates their involvement in the fundamental development of germ cells during spermatogenesis. We concluded that the PRAME proteins may play multifaceted roles in the nucleus and cytoplasm of germ cells. In addition, the experimental results and discussion presented in this work will lay a foundation for the future investigation on the molecular mechanism underlying the function(s) of the PRAME protein family during spermatogenesis.

## Data Availability

All data generated or analyzed during this study are included in this published article.

## Abbreviations

AC: Acrosome
AG: Acrosome granule
AM: Acrosome matrix
AP: Acroplaxome
AV: Acrosome vesicle
CB: Chromatoid body
CD: Cytoplasmic droplet
Chr: Chromosome
cKO: Conditional knockout
CP: Connecting piece
CT: Cancer-testis
CTA: Cancer/testis antigen
DDX4: DEAD-box helicase 4
EP: End piece
ER: Endoplasmic reticulum
ESC: Embryonic stem cell
FITC: Fluorescein isothiocyanate
hPRAME: Human PRAME
IAM: Inner acrosome membrane
IEM: Immunogold electron microscopy
IF: Immunofluorescence
IMC: Intermitochondrial cement
IP: Implantation plate
IS: Implantation socket
KIF17b: Kinesin family member 17b
KIFC1: Kinesin family member C1
KIFs: Kinesin superfamily proteins
LRR: Leucine-rich repeat
M or Mit: Mitochondria
MAEL: Maelstrom spermatogenic transposon silencer
MAG: Mitochondria-associated granule
Man: Manchette
MHV: Mouse homolog of vasa
MP: Midpiece
N: Nucleus
NE: Nuclear envelope
NFY: Nuclear transcription factor Y
NLS: Nuclear localization signal
No: Nucleolus
NP: Nuclear pore
PBS: Phosphate-buffered saline
PBST: PBS with Tween 20
PFA: Paraformaldehyde
PIWIL1: Piwi like RNA-mediated gene silencing 1
PNR: Perinuclear ring
PP: Principal piece
PRAME: Preferentially expressed antigen in melanoma
Pramef12: Prame family 12
PRAMEL1: Prame like 1
Pramel7: Prame like 7
PRAMEX1: Prame, X-linked 1
RA: Retinoic acid
RAR: Retinoic acid receptor
RBPs: RNA binding proteins
RNP: Ribonucleoprotein
SCO: Sertoli cell-only
SSC: Spermatogonial stem cell
SV: Small vesicles
TDRD1: Tudor domain containing protein 1
TGN: Trans-Golgi network
TRITC: Tetramethylrhodamine
